# Some Questions About Herbal and Traditional Medicine

**DOI:** 10.17795/nmsjournal18079

**Published:** 2014-04-17

**Authors:** Efat Aminolroayaee Yamini

**Affiliations:** 1 Kashan's Department of Education, Kashan, IR Iran

**Keywords:** Herbal Medicine, Medicine, Traditional, Complementary Therapies

Dear Editor,

In recent years we have witnessed the widespread use of herbal and traditional medicines. In many cases, people use traditional treatments arbitrarily and without consultation with a qualified person (1). As a teacher, I have frequently seen a number of my colleagues and others use herbal medicines and traditional methods for their medical problems. Looking at the papers published in this journal, it could be inferred that nurse researchers have a particular interest in the impact of herbal, traditional and complementary medicine on the care and treatment of certain physical and psychological problems. For example Sodouri et al. studied the effect of *Zataria multiflora* on the severity of premenstrual syndrome and reported that this herb was not effective on premenstrual pain (2). In addition, Vakilian and Keramat examined the effect of lavender on the first and second stages of labor. They reported that lavender essential oil aroma could be used to decrease the length of a delivery (3). A number of other studies have examined the effects of methods such as, massage therapy and acupressure, on vital signs and blood cortisol levels in patients with heart disease (4, 5), and surgical patients (6), and they reported that these methods were useful. 

Nurses desire to study the effects of herbal medicine and traditional treatments is evident in that such articles can also be found abundantly in other Iranian journals (7-9). Moreover, an Iranian nursing journal has been named, 'Complementary Medicine Journal of Faculty of Nursing and Midwifery'. Although it represents an interest in the development of health science through an approach based on national traditional medicine, questions remain concerning the extent to which the use of herbal medicines and traditional treatments are effective, in comparison with current modern medicine. Are the dosage, frequency of use, and adverse effects, of herbal medicines and traditional treatments as clear as with drugs that are commonly used in modern medicine? Furthermore, can herbal and traditional methods be used alone or should they stand alongside conventional treatments?
